# Self-perceived dysphagia in non-invasively ventilated COVID-19 patients

**DOI:** 10.1007/s00405-022-07557-7

**Published:** 2022-08-10

**Authors:** Mariam S. Shadi, Mohamed Farahat

**Affiliations:** 1 Department of Otorhinolaryngology, Unit of Phoniatrics, Faculty of Medicine, Ain Shams University, Cairo, Egypt; 2 Department of Otolaryngology, Research Chair of Voice, Swallowing, and Communication Disorders, Head and Neck Surgery, College of Medicine, King Saud University, Riyadh, Saudi Arabia

**Keywords:** COVID-19, Dysphagia, Swallowing, EAT-10

## Abstract

**Purpose:**

COVID-19 is known to present with a wide range of clinical symptoms. COVID-19-related dysphagia has been frequently investigated in patients who were critically ill and mechanically ventilated, but not in those with less severe presentations. This study aims to identify the frequency, characteristics, and severity of self-perceived oropharyngeal dysphagia in non-intubated COVID-19 patients.

**Methods:**

In this cross-sectional study, data were collected from patients using a self-administered questionnaire that included the Eating Assessment Tool (EAT-10).

**Results:**

The study included 359 participants with a median age of 34 (range: 18–65) years. Self-perceived dysphagia (EAT-10 total score > 2) was identified in 64.62%, and their median EAT-10 total score was 13 (range 3–40). The most prevalent symptoms were painful swallowing, affected pleasure of eating, stressful swallowing, and coughing while eating. Age, gender, and hospitalization were not statistically significantly associated with the presence of dysphagia, while re-infection, duration, and severity of COVID-19 diagnosis were. The EAT-10 total score was higher in moderate and severe COVID-19 cases as compared to mild cases, and showed a statistically significant inverse correlation with the duration of COVID-19 (*r* = − 0.267).

**Conclusion:**

Self-perceived dysphagia was prevalent in non-intubated COVID-19 patients. Its severity was related to that of COVID-19 and its duration.

## Background

Severe acute respiratory syndrome Coronavirus 2 (SARS-CoV-2), referred to as coronavirus disease 2019 (COVID-19), is primarily a viral respiratory illness, nevertheless it influences other body systems [1–3], and its presentation is now known to be extremely heterogeneous with diverse clinical manifestations [3].

Patients with symptoms can range from mild to life-threatening critical clinical status [4]. The duration of symptoms is influenced by the severity of the condition, which can be related to age and comorbidities [5], with symptoms persisting for up to 2 weeks in mild cases and up to 6 weeks in severe cases [6]. Most COVID-19 cases develop only mild illness [7]. The China Disease Control and Prevention Center evaluated around 44,500 confirmed cases. They reported that 81% of patients had mild (no pneumonia or mild pneumonia), 14% had severe (e.g., dyspnea, hypoxia, or developing more than 50% lung involvement in imaging within 24–48 h), and 5% had critical disease (e.g., respiratory failure, shock, or multiorgan dysfunction requiring intensive care unit (ICU) admission [8].

Several authors attempted to postulate possible explanations for the dysphagia pathogenesis in COVID-19. These may not only be attributed to ICU stays and invasive ventilatory support, but also to factors specifically related to the disease process itself and its complications [4]. Since swallowing and respiration are closely linked, both anatomically and physiologically [4], the synchronization of laryngeal closure, apnea period, and swallowing is crucial to airway protection. Thus, a major cause of dysphagia in patients with respiratory compromise, including COVID-19 patients, is the disrupted breathing pattern and *swallowing-breathing incoordination* [4]. Current evidence also supports the presence of *neurological impairment* in COVID-19, and dysphagia may be one possible consequence. SARS-CoV-2 infection may disrupt the complex neural network that carries out and coordinates the act of swallowing, impacting sensory and motor processes associated with deglutition [9, 10]. To enter the target cells, SARS-CoV-2 is believed to engage angiotensin-converting enzyme 2 (ACE2) receptors and use transmembrane protease serine 2 (TMPRSS2) expressed in different tissues, including the olfactory and gustatory epithelia, the pharynx, larynx, and vocal folds [10]. The virus is thought to penetrate through peripheral nervous endings and subsequently moves in a retrograde trans-neuronal pathway to reach the cranial nerves and the central nervous system [11]. SARS-CoV-2 viral invasion of cranial nerves can cause loss of smell (anosmia) and taste changes (dysgeusia) in mild and moderate COVID-19 [6, 7], as well as sensory changes in swallowing-related areas [9, 10, 12]. *Reduced smell and taste* impair adequate salivation, bolus preparation, oropharyngeal readiness, swallowing efficiency, and meal enjoyment [4, 10], and *decreased pharyngolaryngeal sensation* may jeopardize swallowing efficiency and safety [10, 13]. Moreover, *changes in the muscles of swallowing,* e.g., the tongue, may also induce dysphagia. Skeletal muscle loss of 0.5–6% per day may accompany any mild illness due to the associated inactivity [14]. Myalgia and muscle wasting are well-recognized symptoms of COVID-19 [15]. Elevated serum creatine kinase levels [16], sarcopenia [14], and viral-induced myositis [14, 17] have also been linked to COVID-19. In addition, *prolonged non-invasive respiratory support* implemented in connection with severe COVID-19 pneumonia, such as continuous positive airway pressure (CPAP) or high-flow nasal oxygen (HFNO) therapy, may further compromise swallowing safety. The steady positive pressure of HFNO can adversely affect the pharyngeal and laryngeal chemo- and mechanoreceptors, resulting in hypoesthesia, delayed triggering of the swallow reflex, uncoordinated breathing-swallowing pattern, and a weak cough reflex [18].

Dysphagia is a common complication in post-ICU patients in general [19]. High incidence of dysphagia in COVID-19 patients who were hospitalized, treated in the ICU, or mechanically ventilated has also been reported [e.g., 2, 13, 20, 21].

In clinical practice, we have seen COVID-19 patients with mild or moderate symptoms, or who were admitted to hospital wards and received a ventilatory assistance that was non-invasive in nature, and were complaining of swallowing problems. Nonetheless, there is a paucity of information about the prevalence and patterns of dysphagia in this group of patients.

This study aims to estimate the frequency of self-perceived oropharyngeal dysphagia and characterize its features and severity, using the Arabic EAT-10 tool, in a cohort of COVID-19 patients who were non-intubated i.e., have not been treated with invasive ventilation, in order to give a valuable insight into the effects of SARS-CoV-2 infection on the swallowing function. Sharing clinical experiences would contribute to supplementing the available evidence in this minimally published topic.

## Patients and methods

### Patients

Patients who were recently diagnosed with COVID-19 based on a nasopharyngeal swab and a polymerase chain reaction (PCR) test (whether symptomatic or not) as they visited the University Hospital COVID-19 Outpatient Clinic for diagnosis or follow-up were included. Exclusionary criteria were age below 18 or above 65 years, critically-ill cases, who received invasive ventilatory support or non-oral feeding, impaired level of consciousness, inability to fill in the questionnaire themselves, previous diagnosis, signs or symptoms of dysphagia, history of a neurological insult/disease, with or without a history of dysphagia, previous head, and neck, esophageal, stomach, lung cancer or radiotherapy, or other medical/surgical conditions that might potentially affect the swallowing mechanism. Patients with a SARS-CoV-2 re-infection were included, provided that they had no history of swallowing symptoms before the current illness.

### Methods

To verify eligibility criteria and collect the intended information, recruited patients filled in a questionnaire, using either a printed form or an online Google form. Questionnaires were distributed and collected by the same nurse.

### Study design

Observational cross-sectional study.

### Data collection for patient characteristics

Data collection took place in the period between January and October 2021. Patient characteristics included age, gender, how and when the COVID-19 diagnosis was established, if the current illness was the first-ever diagnosed COVID-19, the severity of infection, and hospitalization. The severity of COVID-19 was primarily determined by participants, guided by the listed clinical manifestations. Health care providers were also available should patients need additional counseling to rate their disease severity. The WHO [7] clinically categorized COVID-19 as follows:—*Mild illness:* uncomplicated upper respiratory tract viral infection, with mild, non-specific symptoms such as fever, fatigue, cough (with or without sputum production), anorexia, malaise, muscle pain, sore throat, dyspnea, nasal congestion, headache, diarrhea, nausea, or vomiting.—*Pneumonia:* pneumonia is not severe, no need for supplemental oxygen.—*Severe pneumonia:* radiological findings of severe pneumonia, fever, or suspected respiratory infection, plus one of the following: respiratory rate > 30 breaths/min, severe respiratory distress, or oxygen saturation of ≤ 93% on room air.—*Acute respiratory distress syndrome (ARDS)*, *Sepsis, or Septic shock.* Based on the WHO description [WHO interim guidance], cases were categorized as mild, moderate, or severe if they presented with symptoms of ‘mild illness’, ‘pneumonia’, or ‘severe pneumonia’ respectively.

### Outcome measure

Self-perceived swallowing outcomes were obtained from the Arabic Eating Assessment Tool (EAT-10) [22] as part of the questionnaire. It comprises 10 statements, each rated on a 5-point scale of 0 to 4, with a summated score that ranges from 0 to 40. A total score of > 2 is considered abnormal [23] and the higher the score, the greater the dysphagia it indicates.

The EAT-10 is a simple, easily read and understood [24], valid and reliable [22] tool. It is non-invasive, self-administered [22], and patient-reported, so it best reflects the patient-centered subjective impression of swallowing difficulties, and it is particularly encouraged in cases with confirmed COVID-19 [25], as it carries a very minimal risk of viral spread, if any. It is also symptom-specific and it is utilized to determine the severity of self-perceived dysphagia [24].

Optimally, EAT-10 should be used to identify persons at risk of dysphagia and refer them for an additional swallowing evaluation. However, given the constraints mandated by the current pandemic context, such as the limitation of many swallowing screening tools [26] and the suspension of non-urgent instrumental assessments for a potential aerosol generation [27], this was not feasible, especially that COVID-19 patients are more liable to cough during the procedure due to their concomitant pulmonary condition.

### Infection control

Patients completed the questionnaires themselves, whenever possible. Using the soft copy version of the questionnaire has always been encouraged. Paper forms were collected and stored in a designated box for at least 3 weeks before being processed for data entry.

### Dealing with missing data

When a received questionnaire was incomplete, the missing fields were filled out by contacting the respondent. If this was not feasible or if any single value remained missing, the entire record was excluded.

### Statistical methods

The Statistical Package for Social Science (SPSS), version 26 (IBM Corp., Armonk, NY, USA) was used to analyze the data. The normality of the data was tested using the Kolmogorov–Smirnov single-sample test. Numerical data were presented as mean and standard deviation, or median and range. Comparison between two groups for numerical variables was done using the student *t*-test. Comparison between more than two groups for numerical variables was done using the Kruskal–Wallis followed by a post hoc test for pairwise comparison between groups. Continuous data were correlated using Spearman correlation. Qualitative data were described as frequency and percentage. The Chi-square or Fisher’s exact test was used to examine the relationship between qualitative variables as appropriate. All tests were two-tailed. A *p*-value of < 0.05 was considered statistically significant.

## Results

Data from 456 respondents were collected; 97 were excluded, and the remaining 359 were considered for statistical analysis. Excluded responses included incomplete ones (*n* = 31), repeated entries on electronic forms for the same patient (*n* = 7), and patients who had not met the selection criteria (*n* = 59). The clinical and demographic characteristics of patients are summarized in Table 1.

**Table 1:** Clinical and demographic characteristics of the study population (*n* = 359)

	Mean ± SD	Median (range)
Age (years)	35.92 ± 10.05	34 (18–65)

	*n*	%
Gender		
Male	129	35.9
Female	230	64.1
Time from COVID-19 diagnosis		
1 week or less	235	65.5
2 weeks	26	7.2%
3 weeks	22	6.1
4 weeks	9	2.5
5 weeks or more	67	18.7
First time to contract COVID-19 infection		
Yes	265	73.8
No	94	26.2
Hospitalization: (no invasive ventilation)		
Yes	22	6.1
No	337	93.9
Severity of COVID-19 infection:		
Asymptomatic	4	1.1
Mild	218	60.7
Moderate	115	32.0
Severe	22	6.1

*SD* standard deviation, *n.* number, *%* percentage

**Fig. 1:**
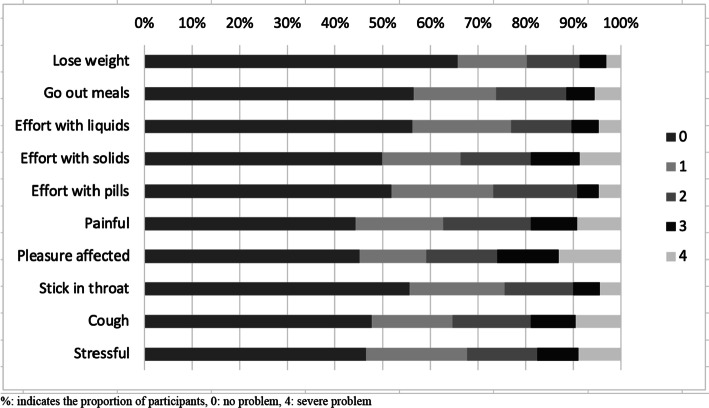
Proportions of participants’ scores in each of the individual items of the EAT-10, *n* = 359

Participants’ self-perception of the presence of swallowing problems showed that 232 patients (64.62%, 95% confidence interval: 59.58–69.44%) had dysphagia (Table 2). The proportions of participants’ scores in each of the individual items of EAT-10 are illustrated in Fig. 1.

**Table 2:** Frequency of dysphagia among participants as well as their EAT-10 total score

	All participants	Patients without dysphagia	Patients with dysphagia^a^
n (%)	359 (100%)	127 (35.38%, 95% CI 30.56–40.42%)	232 (64.62%, 95% CI 59.58–69.44%)
EAT-10 total score median (range)	8 (0–40)	0 (0–2)	13 (3–40)

*n* number, *%* percentage, *SD* standard deviation, *CI* confidence interval

^a^Self-perceived dysphagia identified by an EAT-10 total score > 2

The most frequent self-perceived dysphagia symptom was ‘painful swallowing’ (*n* = 200, 55.7%), followed by ‘swallowing problem affected the pleasure of eating’ (*n* = 197, 54.9%), stressful swallowing (*n* = 192, 53.5%) ‘coughing upon eating’ (*n* = 188, 52.4%), ‘swallowing solids took extra effort’ (*n* = 180, 50.1%), ‘swallowing pills took extra effort’ (*n* = 173, 48.2%), ‘food stuck in the throat during swallowing’ (*n* = 159, 44.3%), ‘swallowing liquids took extra effort’ (*n* = 157, 43.7%), ‘swallowing problem interfered with the ability to go out’ (*n* = 156, 43.5%), with ‘swallowing problem caused weight loss’ being the least frequent (*n* = 123, 34.3%).

The relationships between self-perceived dysphagia, its presence, and severity (as reflected by the EAT-10 total score), and the independent variables were studied. Age, gender, and hospitalization were not statistically significantly associated with the presence of dysphagia, while re-infection, duration, and severity of COVID-19 diagnosis were (Table 3). Dysphagia was more common in individuals who had COVID-19 for the second time (79%) than in those who had COVID-19 for the first time (60%) (Table 3).

**Table 3:** Relation between the presence of dysphagia symptoms (EAT-10 total score > 2) and patient characteristics

	Dysphagia^a^				*p* value
	No		Yes		
	Mean	SD	Mean	SD	
Age (years)	37.23	10.58	35.21	9.72	0.068

	*n*	%	*n*	%	
Gender					
Male	44	34.1%	85	65.9%	0.707
Female	83	36.1%	147	63.9%	
Time from COVID-19 diagnosis					
1 week or less	61	26.0%	174	74.0%	< 0.001^«^
2 weeks	12	46.2%	14	53.8%	
3 weeks	13	59.1%	9	40.9%	
4 weeks	4	44.4%	5	55.6%	
5 weeks or more	37	55.2%	30	44.8%	
First time to have COVID-19:					
Yes	107	40.4%	158	59.6%	0.001^«^
No	20	21.3%	74	78.7%	
Hospitalization:					
Yes	6	27.3%	16	72.7%	0.412
No	121	35.9%	216	64.1%	
Severity of COVID-19 infection:					
Asymptomatic	4	100%	0	0.0%	0.001^«^
Mild	88	40.4%	130	59.6%	
Moderate	30	26.1%	85	73.9%	
Severe	5	22.7%	17	77.3%	

*SD* standard deviation, *n.* number,* %* percentage

^«^Significant

^a^Self-perceived dysphagia identified by an EAT-10 total score > 2

When assessed during the first week of diagnosis, about 3/4 of patients experienced self-perceived dysphagia, and the proportion statistically significantly declined when patients were assessed in the following weeks (Table 3). Furthermore, the length of time since the COVID-19 diagnosis showed a weak yet statistically significant inverse correlation with the severity of dysphagia (*r* = − 0.267, *p* < 0.001). On the other hand, age was not statistically significantly correlated to dysphagia symptoms severity (*r* = − 0.083, *p* = 0.117).

The frequency of dysphagia was statistically significantly higher in patients with a more severe illness than in those with a milder illness (Table 3). Furthermore, severe and moderate COVID-19 cases had a statistically significantly (*p* < 0.001) higher EAT-10 score than mild cases, indicating a more severe self-perceived dysphagia, but no statistically significant difference was found between ‘moderate’, and ‘severe’ cases. The median (range) EAT-10 scores of ‘asymptomatic and mild’, ‘moderate’, and ‘severe’ COVID-19 cases were 5 (0–33), 12 (0–40), and 14 (0–40) respectively.

## Discussion

### Summary of results

This study describes the self-perceived deglutition function in 359 patients diagnosed with COVID-19, who have been treated either at home or in hospital wards but have neither been critically ill nor required invasive ventilation. Dysphagia was identified in nearly two-thirds of patients, with a median EAT-10 score of 13 (range 3–40). The frequency of each problem of the 10 items of EAT-10 ranged between 55.7% and 34.2%. The presence of dysphagia was associated with a more severe COVID-19 illness, a shorter duration from its diagnosis, or a re-infection. The severity of dysphagia was greater in more severe COVID-19 cases and was inversely correlated to the time elapsed since the COVID-19 diagnosis.

### Previous studies

To the authors’ knowledge, only one study [4], published prior to the current one, entirely focused on dysphagic COVID-19 patients who were not invasively ventilated. Eight (19.5%, 95% CI 10–34%) out of 41 non-intubated patients exhibited dysphagia symptoms during hospitalization. Having said that, it is important to note that their dysphagia symptoms evaluation was made at a different time point. Those patients have already overcome their acute illness and were asymptomatic, although still positive for SARS-CoV-2 in the PCR test, whereas the majority of our participants were evaluated during their acute phase of COVID-19. Furthermore, contrary to Grilli et al. [4], we excluded patients with chronic obstructive pulmonary disease (COPD) and bronchial asthma since there is a recognized correlation between pre-existing respiratory diseases and oropharyngeal dysphagia that has also been noted in non-intubated COVID-19 patients in particular [4].

Similarly, one study [12] involved COVID-19 patients with mild (71.5%) and moderate (28.4%) severity and showed that 24 (20.6%) out of 116 patients suffered from dysphagia. Another study [19] found that 3 (14%) of the small preliminary dataset of 21 non-hospitalized patients presented with self-reported dysphagia. The disparity between the results of these studies and ours could be largely explained by the differences in sample size.

### Analysis of the EAT-10 results

The swallowing problems that were most frequently encountered by patients were: painful swallowing, affected pleasure of eating, stressful swallowing, and coughing when eating. *Painful swallowing* (odynophagia) was most likely caused by pharyngitis and sore throat symptoms, a well-recognized early COVID-19 complaint [6, 12].

The *affected pleasure of eating* could be attributed to swallowing impairment, but can also be due to or aggravated by the concomitant smell and taste affection. Although not particularly evaluated in this study, olfactory and gustatory impairment are among the most common symptoms of COVID-19 [28], especially in cases with a milder clinical course [29], which accounts for the vast majority of our cases.

As coughing is a chief COVID-19 symptom across the different severity categories [30], we may assume that patient-reported *coughing while eating* was either directly related to the pulmonary condition caused by COVID-19 or to the accompanying dysphagia and aspiration. Even without the instrumental visualization of swallowing, observing the frequency and timing of cough attacks in relation to meals can help distinguish between both origins of cough. Several authors argued that the subjective impression of dysphagia measured by the EAT-10 can identify dysphagic patients with inadequate airway protection [31, 32], with a total score of more than 15 increasing the risk of aspiration by 2.2 times [32]. The median EAT-10 total score for participants with identifiable dysphagia was less than this cut-off point, as well as less than the mean for patients with oropharyngeal dysphagia (23.10 ± 12.22) in the study by Belfasky et al. in 2008 [23]. However, given the frequency of other dysphagia symptoms we found in COVID-19 patients, aspiration can still be an expected event. In COVID-19 patients, aspiration associated with swallowing difficulties may be an additional risk factor for developing superimposed pneumonia, worsening their pulmonary condition [1, 3, 4].

Patients reported *extra effort needed to swallow liquids, solids, and pills, with a feeling of food getting stuck in the throat*. This may point out a propulsion deficit, likely a consequence of viral neural complications, as dysphagia to both solid and liquid boluses is primarily associated with oral, pharyngeal, and laryngeal motility ailments [33]. It is important to note, though, that this explanation is just an assumption that needs to be verified instrumentally.

*Interference with the ability to go out for meals due to the swallowing problem* was a less frequent problem. Although this item did not solely reflect the handicap imposed by dysphagia, since it could also have been influenced by the mandatory quarantine for COVID-19 cases, this did not affect the study’s findings.

*Losing weight due to the swallowing problem* was the least prevalent symptom among participants. This is most likely because the majority of participants had mild or moderate diseases that typically lasted less than 2 weeks [6], as well as the fact that data was primarily collected (in 65.5% of cases) during the first week of illness. In the case of persistent dysphagia symptoms, it is possible that weight loss would have become more evident as time elapses.

The short duration of illness can explain why the swallowing problems have not resulted in weight loss in all patients. Nevertheless, nearly one-third of participants reported *losing weight.* It can be attributed not only to the swallowing problem, but also to the accompanying COVID-19-related decreased/loss of appetite (hyporexia/anorexia) [14], changes in smell and taste, or gastrointestinal symptoms [34]. Anorexia may be related to COVID-19 symptoms, including smell and taste affection, cough, dyspnea, or other manifestations of acute infection [13]. Anorexia in COVID-19 patients lasts around 7 ± 10 days [35], and can result in malnutrition and secondary sarcopenia, which in turn increases the risk of dysphagia and delays recovery [3].

### Pathogenesis of dysphagia in COVID-19

Suggested theories of swallowing breakdown in COVID-19 patients have been discussed earlier. However, it should be noted that the majority of published studies were concerned with ICU-admitted patients, rather than the less severe cases as in our study. Logically, the complications associated with milder infections may not be as cruel as those in more severe ones. For instance, respiratory-swallowing asynchronization associated with ARDS in critically ill cases is expected to be more aggressive than that associated with moderate cases where pneumonia is not severe.

### Association of dysphagia severity with other variables

Old age was usually linked to greater ICU admission rates and mortality from COVID-19, particularly among those with comorbidities [19]. In the current study, the average age of participants happened to be in the mid-30 s, probably because we included less severe, non-ICU patients. *Age* was neither statistically significantly associated with the presence of dysphagia nor with its severity. A similar observation was reported by Özçelik Korkmaz et al. [12] who found no statistically significant difference between age and the clinical severity of COVID-19. Grilli et al. [4] observed that patients with dysphagia were younger, leading them to think that COVID-19 was the primary cause of swallowing difficulties in their study sample.

The inverse correlation between dysphagia severity and the elapsed *time since the COVID-19 diagnosis* may suggest that self-perceived dysphagia may gradually fade over time. This is consistent with results from earlier studies showing that self-reported dysphagia symptoms in non-intubated COVID-19 patients tended to spontaneously resolve in most cases. In the study by Grilli et al. [4], dysphagia was spontaneously resolved in six out of eight patients who initially demonstrated dysphagia during hospitalization. Their observations need to be confirmed with a larger sample, though. Özçelik Korkmaz et al. [12] showed that the median dysphagia duration in mild and moderate COVID-19 patients, assessed over a period of 30 days, was 5 days (range: 1–13 days).

The *severity of the COVID-19 illness* was directly correlated to the severity of dysphagia in a statistically significant manner. It is reasonable to think that severe cases of an infection are more likely to experience more aggressive complications, and COVID-19 is not an exception. Additionally, non-invasive ventilation treatments, e.g., CPAP or HFNO, may be required in severe COVID-19.

Furthermore, the observation that *hospitalization* was not associated with a higher degree of dysphagia severity, yet the severity of COVID-19 was, could be explained by the fact that not all hospitalized patients were necessarily the most severe cases among participants; some severe patients were treated at home while other moderate cases were treated at hospital wards.

The finding that the proportion of dysphagia in patients who contracted *COVID-19 for the first time* was less than that in those who had a second infection may suggest that a past SARS-CoV-2 infection makes patients more vulnerable to developing dysphagia upon re-infection, even if the previous one has not resulted in any swallowing impairment.

### Limitations

The current study has some limitations. Firstly, due to the current pandemic situation, infection control restrictions, staff shortages, and pressurized healthcare systems, the lack of instrumental dysphagia evaluation may be considered a contextual rather than a design-related limitation. Despite the valuable information obtained from EAT-10 as a patient-reported outcome tool, complementing dysphagia evaluation with further clinical and instrumental measures would have added to the diagnostic competency and understanding of the swallowing mechanism breakdown. Secondly, this is a single-center observational study and should thus be viewed as preliminary data.

### Recommendations and future plans

This study underscores the high prevalence of self-perceived dysphagia in mild and moderate COVID-19 patients, and the importance of exploring such swallowing difficulties in this frequently overlooked group of patients. Following up on these patients would be valuable to understand the fate and evolution of symptoms over time. Re-evaluation of patients should extend beyond recovery from COVID-19 acute illness because prolonged sequelae as well as relapsing–remitting symptom patterns are possible [19].

## Data Availability

The datasets used and analyzed during the current study are available from the corresponding author on reasonable request.
